# Effect of *L. usitatissimum* (Flaxseed/Linseed) Fixed Oil against Distinct Phases of Inflammation

**DOI:** 10.1155/2013/735158

**Published:** 2013-02-17

**Authors:** Gaurav Kaithwas, Dipak K. Majumdar

**Affiliations:** ^1^Department of Pharmaceutical Sciences, Babasaheb Bhimrao Ambedkar University, Raebareli Road, Vidyavihar, Lucknow, Uttar Pradesh 226 025, India; ^2^Department of Pharmaceutical Sciences, Faculty of Health and Medical Sciences, Allahabad Agricultural Institute-Deemed University, Allahabad, Uttar Pradesh 211 007, India; ^3^Delhi Institute of Pharmaceutical Sciences and Research, (Formerly College of Pharmacy), University of Delhi, Sector III Pushp Vihar, New Delhi, Delhi 110 017, India

## Abstract

The present investigation summarizes the effect of *Linum usitatissimum* fixed oil against different phases of acute inflammatory reaction, namely, protein exudation, peritoneal capillary permeability, and leukocyte migration. The fixed oil exhibited dose-dependent inhibition of protein exudation vascular permeability, comparable to standard aspirin. The oil also inhibited the leukocyte migration in pleural exudates in a dose-dependent manner. Production of less vasodilatory (PGE_3_) and chemotactic (LTB_5_) eicosanoids through EPA (derived from linolenic acid) metabolism could account for the above observations.

## 1. Introduction

*Linum usitatissimum *L, (also known as common flax or linseed) an annual herb believed to have originated in Egypt, is a member of the genus *Linum* in the family Linaceae. The seeds produce a fixed oil known as linseed oil or flaxseed oil. It is one of the oldest commercial oils, and solvent processed flaxseed oil has been used for centuries as a drying oil in painting and varnishing. Raw oil is used as an astringent in fungicidal lotion and as an insecticide and has moderate insect repellent properties [1]. The oil contains unsaturated fatty acids like oleic acid (12–30%), linoleic acid (8–29%), and linolenic acid (35–67%) [2]. These fatty acids appear to render drying property to the oil. In earlier studies, the *L. usitatissimum *fixed oil has been reported to exhibit significant anti-inflammatory [3], antiarthritic [4], antiulcer [5], and antidiabetic [6] properties along with the efficacy against experimental esophagitis in experimental animals [7]. The antimicrobial activity of *L. usitatissimum *oil and its therapeutic efficacy in bovine mastitis, an inflammatory disorder caused by microbial infection, has been reported recently [8]. Considering the significant anti-inflammatory and antiarthritic activity, the present study has been undertaken to evaluate the possible effect of fixed oil against distinct phases of inflammation using suitable animal models.

## 2. Materials and Methods

### 2.1. Materials

#### 2.1.1. Plant Material

Flaxseed/linseed (Variety: JL-59) was obtained from The Division of Seed Science, Department of Agronomy, Allahabad Agricultural Institute-Deemed University (AAI-DU), Allahabad, India. The seeds were authenticated at The National Botanical Research Institute (NBRI, CSIR), Lucknow, India, and the voucher sample was deposited at NBRI.

#### 2.1.2. Extraction of Oil

Seeds were crushed and cold macerated in petroleum ether (40–60°C) for 7 days. Petroleum ether was evaporated from the extract, and the oil was filtered to clarity. The oil was stored at room temperature in amber colored airtight bottle. To avoid oxidation, the oil was purged with nitrogen and was filled to the brim of the bottle so that there was no headspace. The yield of fixed oil was 17.50% v/w with reference to dried seeds. The density of the oil was 0.952 g/mL. Gas chromatographic analysis (Agilent GC make 6890; column: DBFFAP, dimension (30 M × 0.53 mm × 1.0 *μ*m) using flame ionization detector; carrier gas: nitrogen; volume of injection 1 *μ*L; internal standard: cetyl alcohol) of the methyl ester of oil revealed the presence of palmitic acid (5.53%), stearic acid (4.67%), oleic acid (19.05%), linoleic acid (13.67%), and linolenic acid (57.38%). The oil thus obtained was subjected to further studies.

#### 2.1.3. Animals

Wistar strains of albino rats (100–150 g) and Swiss strain of albino mice (25–50 g) were obtained from The Central Animal House, Department of Animal Husbandry, Allahabad Agricultural Institute-Deemed University. The animals were housed under standard conditions of temperature (25 ± 1°C) with 12 h light/dark cycle and had free access to commercial pellet diet and water. The animals were given week's time to get acclimatized to the laboratory condition, before experimentation. All experiments were performed according to the CPCSEA guidelines for the laboratory animals and ethics, Department of Animal Welfare, Government of India.

#### 2.1.4. Drugs and Chemicals

Pentobarbitone was purchased from Sigma-Aldrich, USA, and chlorpromazine (Megatil) was procured from Intas Pharmaceuticals Ltd., India. Aspirin and phenylbutazone were received as a generous gift samples from Arbro Pharmaceuticals Ltd., New Delhi, India. All other chemicals were of analytical grade.

### 2.2. Methods

#### 2.2.1. Inflammatory Exudation (Protein Exudation)

Group of albino mice were treated either with *L. usitatissimum *fixed oil (1–3 mL/kg) or control vehicle (distilled water, 3 mL/kg) or standard drug (aspirin, 100 mg/kg) intraperitoneally. After 30 min, 0.25 mL of 1.2% acetic acid was administered i.p. to each mouse. After 3 h, viscera's were exposed by abdominal incision; abdomen was irrigated with physiological saline containing 0.1 mM disodium EDTA. The pooled washing was made to 6 mL with physiological saline and then centrifuged. Total amount of protein in the supernatant was estimated with Folin's reagent [9].

#### 2.2.2. Peritoneal Capillary Permeability (Dye Leakage Method)

*L. usitatissimum *fixed oil (1–3 mL/kg) or control vehicle (distilled water, 3 mL/kg) or standard drug (aspirin, 100 mg/kg) was administered intraperitoneally to group of albino mice (*n* = 6), and after 3 h, each mouse received 4 mL of 0.05 N acetic acid in 0.9% saline i.p. followed by immediate i.v. administration of 0.1 mL of 4% pontamine sky blue (dye). One hour after the administration of the dye, animals were sacrificed by cervical dislocation and the peritoneal cavity was drained. The collected exudate was centrifuged for 10 min, at 2000 rpm, and 0.5 mL of the supernatant was diluted with 4.5 mL of physiological saline. Absorbance of the diluted supernatant was measured in a colorimeter at 625 nm using normal saline as blank. The mean percentage of the light absorption was then calculated for both control and drug-treated groups [10].

#### 2.2.3. Leucocyte Migration

Carrageenan (0.1 mL of 1% aqueous suspension) was injected into the pleural cavity of anesthetized rats as described previously [11], pretreated i.p. 30 min earlier with *L. usitatissimum *fixed oil (1–3 mL/kg) or control vehicle (distilled water, 3 mL/kg) or standard drug (phenylbutazone, 100 mg/kg). After 5 h, the animals were sacrificed and 2 mL of Hank's solution [12] was injected into the pleural cavity. The animals were then rocked to allow the mixing of the solution with the exudates, which was then collected with a pipette. The total number of leucocytes present in the exudate was counted with a haemocytometer [13].

#### 2.2.4. Statistical Analysis

All the data are presented as mean  ± SEM and analyzed by one-way ANOVA followed by Dunnett's test for the possible significance identification between the various groups. *P* < 0.05 was considered statistically significant. Statistical analysis was carried out using Graph padprism 3.0 (Graph pad software, San Diego, CA).

## 3. Results and Discussion

*L. usitatissimum *fixed oil demonstrated a significant (*P* < 0.05) dose-dependent inhibition of protein exudation (i.e., rise in protein concentration in peritoneal fluid) and inhibited the vascular permeability evidenced by inhibition to dye leakage. Degree of inhibition of protein exudation and vascular permeability was comparable to standard aspirin (Table 1). Similarly, in carrageenan-induced pleurisy in rats, *L. usitatissimum *fixed oil demonstrated a significant (*P* < 0.05) dose-dependent inhibition in leucocyte migration in pleural exudates and slightly better results were observed at a dose level of 3 mL/kg (i.p.), in comparison to phenylbutazone (Table 2).

The present investigation revealed the effect of *L. usitatissimum *fixed oil on distinct phases of inflammatory process: firstly an acute phase of local vasodilatation and increased capillary permeability leading to exudation, followed by leucocytes migration. The momentous characteristic of the inflammatory reaction is the transient increase in permeability of small blood vessels to proteins, initially involving the venules, whereas the delayed phase of increased permeability involves the capillaries as well, and is more relevant to chronic inflammation [14]. The effect of *L. usitatissimum *fixed oil on the intensity of peritoneal inflammation was evaluated by direct measurement of exuded plasma protein using Folin's reagent [9] and leakage of protein bound dye into the peritoneal cavity [10]. *L. usitatissimum *fixed oil significantly reduced the peritoneal vascular permeability, indicating the suppression of the vascular response in the process of acute inflammation. Anti-inflammatory potential was further confirmed by inhibition of carrageenan-induced pleurisy in rats, as *L. usitatissimum *fixed oil significantly inhibited the migration of leucocytes in pleural exudates. The key inflammatory mediators are the *n*-6 eicosanoids, prostaglandin E_2_ (PGE_2_), and leukotriene B_4_ (LTB_4_) which are derived from polyunsaturated fatty acid arachidonic acid (AA; 20 : 4, *n*-6) [15] by the action of cyclooxygenase and lipoxygenase. PGE_2_ is a potent vasodilator, whereas LTB_4_ is a powerful chemotactic agent. PGE_2_ does not increase the permeability of postcapillary venules but potentiates the effect of histamine and bradykinin to increase permeability. PGE_2_ also potentiates the effect of bradykinin by sensitising afferent C fibres to increase pain [16]. On the other side, leukotriene (LTB_4_) is a potent chemotactic agent for both neutrophils and macrophages. LTB_4_ binds to its specific surface cell receptors (BLT-1 and BLT-2) and stimulates a number of leukocyte functions, that is, adhesion of neutrophils to vascular endothelium, transendothelial migration, and chemotaxis [17, 18]. Vasodilation and increased capillary permeability permits fluids and large molecules to leave the blood stream and increased movement of leucocytes into the surrounding tissues by diapedesis. Thus, PGE_2_ and LTB_4_ together can cause/promote vascular leakage and extravagation of fluids and are significantly involved in the physiology of acute inflammation [15].

The anti-inflammatory activity of various plant lipids has been reported, and the results revealed that the lipids containing *α*-linolenic acid (ALA, 18 : 3, *n*-3), for example, linseed oil, *O. sanctum *fixed oil, and soyabean oil had a significant anti-inflammatory activity. Subsequently, methyl esters of various fatty acids, namely, oleic acid, linoleic acid, and ALA, were tested against PGE_2_-, LTB_4_, and arachidonic acid-induced paw oedema in rats, where only ALA showed a significant oedema inhibition against all the inflammatory mediators suggesting the ALA as a dual inhibitor of arachidonate metabolism [19]. Previously, we also observed antiarthritic activity of the *L. usitatissimum *fixed oil against turpentine oil-induced joint oedema, formaldehyde-induced and adjuvant-induced arthritis in albino rats [4]. ALA (18 : 3, *n*-3) is a precursor for eicosapentaenoic acid (EPA, 20 : 5, *n*-3), which competes with AA (20 : 4, *n*-6, precursor for lipid mediators of inflammation) for cyclooxygenase and lipoxygenase pathway. EPA by acting as a substrate for cyclooxygenase and lipoxygenase pathway produces PGE_3_ (less potent vasodilator than PGE_2_) [20] and LTB_5_ (100 times less potent chemotactic than LTB_4_) [21–23]. Thus, less vasodilatory (PGE_3_) and chemotactic (LTB_5_) response of lipid mediators derived from EPA (metabolic product of ALA metabolism) accounts for the inhibition of fluid and protein exudation along with diminished leucocytes migration observed in the present experiment.

## Figures and Tables

**Table 1:** Effect of *L. usitatissimum *fixed oil and aspirin on acetic acid induced peritoneal inflammation and peritoneal capillary permeability in mice.

Serial number	Treatment	Dose	Total amount of protein (mg/mL) exudation 3 h after acetic acid administration (0.25 mL of 1.2%, i.p.)	Absorbance at 625 nm
1	Control (distilled water)	3 mL/kg	5.01 ± 0.21	0.49 ± 0.02
2	*L. usitatissimum* fixed oil	1 mL/kg	4.15 ± 0.24* (17.17)	0.39 ± 0.05* (20.41)
3	*L. usitatissimum* fixed oil	2 mL/kg	2.82 ± 0.21* (43.71)	0.31 ± 0.03* (36.73)
4	*L. usitatissimum* fixed oil	3 mL/kg	1.59 ± 0.18* (68.26)	0.19 ± 0.05* (61.22)
5	Aspirin	100 mg/kg	1.57 ± 0.16* (68.66)	0.20 ± 0.03* (59.18)

Values in parenthesis represent percentage inhibition (values are mean ± SEM).

Each group contains six animals; all groups were compared to the group 1 by Dunnett's test (*P < 0.05).

**Table 2:** Effect of *L. usitatissimum *fixed oil and phenylbutazone on carrageenan-induced pleurisy (leucocytes migration) in albino rats.

Serial number	Treatment	Dose	Number of leucocytes (×10^3^/mm^3^)
1	Control (distilled water)	3 mL/kg	8854.25 ± 112.21
2	*L. usitatissimum* fixed oil	1 mL/kg	6524.24 ± 302.14* (26.31)
3	*L. usitatissimum* fixed oil	2 mL/kg	3954.25 ± 124.25* (55.34)
4	*L. usitatissimum* fixed oil	3 mL/kg	3548.45 ± 258.26* (59.92)
5	Aspirin	100 mg/kg	3654.88 ± 222.45* (58.72)

Values in parenthesis represent percentage inhibition (values are mean ± SEM); each group contains six animals; all groups were compared to the control by Dunnett's test (*P < 0.05).

## References

[1] The Wealth of India (2006). *A Dictionary of Indian Raw Materials and Industrial Products*.

[2] The Wealth of India (1976). *Industrial Products, Part IX*.

[3] Kaithwas G, Mukherjee A, Chaurasia AK, Majumdar DK (2011). Antiinflammatory, analgesic and antipyretic activities of *L. usitatissimum* (flaxseed/linseed) fixed oil. *Indian Journal of Experimental Biology*.

[4] Kaithwas G, Majumdar DK (2010). Therapeutic effect of *Linum usitatissimum* (flaxseed/linseed) fixed oil on acute and chronic arthritic models in albino rats. *Inflammopharmacology*.

[5] Kaithwas G, Majumdar DK (2010). Evaluation of antiulcer and antisecretory potential of *Linum usitatissimum* fixed oil and possible mechanism of action. *Inflammopharmacology*.

[6] Kaithwas G, Majumdar DK (2012). In vitro antioxidant and in vivo antidiabetic, antihyperlipidemic activity of linseed oil against streptozotocin-induced toxicity in albino rats. *European Journal of Lipid Science and Technology*.

[7] Renu N, Kaithwas G, Ramteke PW, Saraf SA (2012). Effect of *Linum usitatissimum * (Linseed/Flaxseed) fixed oil on experimental esophagitis in elbino rats. *Acta Gastro-Enterologica Belgica*.

[8] Kaithwas G, Mukerjee A, Kumar P, Majumdar DK (2011). *Linum usitatissimum* (linseed/flaxseed) fixed oil: antimicrobial activity and efficacy in bovine mastitis. *Inflammopharmacology*.

[9] Lowry OH, Rosebrough NJ, Farr AL, Randall RJ (1951). Protein measurement with the Folin phenol reagent. *The Journal of Biological Chemistry*.

[10] Filderman RB, Kovacs BA (1969). Anti-inflammatory activity of the steroid alkaloid glycoside, toatine. *British Journal of Pharmacology*.

[11] Meacock SCR, Kitchen EA (1979). Effects of the non-steroidal anti-inflammatory drug benoxaprofen on leucocyte migration. *Journal of Pharmacy and Pharmacology*.

[12] Cruickshank R (1965). *Medical Microbiology*.

[13] Di Rosa M, Giroud JP, Willoughby DA (1971). Studies on the mediators of the acute inflammatory response induced in rats in different sites by carrageenan and turpentine. *Journal of Pathology*.

[14] Swingle KF, Scherrer RA, Whitehouse MW (1974). Evaluation for antiinflammatory activity. *Antiinflammatory Agents: Chemistry Pharmacology*.

[15] Henderson B, Pettipher ER, Higgs GA (1987). Mediators of rheumatoid arthritis. *British Medical Bulletin*.

[16] Rang HP, Dale MM, Ritter JM, Moore PK (2003). *Pharmacology*.

[17] Yokomizo T, Izumi T, Chang K, Takuwa Y, Shimizu T (1997). A G-protein-coupled receptor for leukotriene B4 that mediates chemotaxis. *Nature*.

[18] Yokomizo T, Kato K, Terawaki K, Izumi T, Shimizu T (2000). A second leukotriene B4receptor, BLT2: a new therapeutic target in inflammation and immunological disorders. *Journal of Experimental Medicine*.

[19] Singh S, Majumdar DK (1997). Evaluation of antiinflammatory activity of fatty acids of *Ocimum sanctum* fixed oil. *Indian Journal of Experimental Biology*.

[20] Hawkes JS, James MJ, Cleland LG (1991). Separation and quantification of PGE3 following derivatization with panacyl bromide by high pressure liquid chromatography with fluorometric detection. *Prostaglandins*.

[21] James MJ, Gibson RA, Cleland LG (2000). Dietary polyunsaturated fatty acids and inflammatory mediator production. *The American Journal of Clinical Nutrition*.

[22] Lee TH, Menica-Huerta JM, Shih C, Corey EJ, Lewis RA, Austem KF (1984). Characterization of biologic properties of 5, 12- dihydroxy derivative of eicosapentaenoic acid, including leukotriene B5 and double lipoxygenase product. *The Journal of Biological Chemistry*.

[23] Goldman DW, Pickett WC, Goetzl EJ (1983). Human neurtrophil chemotactic and degranulating activites of leukotriene B5 (LTB5) derived from eicosapentaenoic acid. *Biochemical and Biophysical Research Communications*.

